# Modulations of bioactive lipids and their receptors in postmortem Alzheimer’s disease brains

**DOI:** 10.3389/fnagi.2022.1066578

**Published:** 2022-12-09

**Authors:** Makoto Kurano, Yuko Saito, Baasanjav Uranbileg, Daisuke Saigusa, Kuniyuki Kano, Junken Aoki, Yutaka Yatomi

**Affiliations:** ^1^Department of Clinical Laboratory Medicine, Graduate School of Medicine, The University of Tokyo, Tokyo, Japan; ^2^Tokyo Metropolitan Geriatric Hospital, Institute of Gerontology, Tokyo, Japan; ^3^Laboratory of Biomedical and Analytical Sciences, Faculty of Pharma-Science, Teikyo University, Tokyo, Japan; ^4^Department of Health Chemistry, Graduate School of Pharmaceutical Sciences, The University of Tokyo, Tokyo, Japan

**Keywords:** Alzheimer’s disease, lipidomics, lysophospholipids, sphingolipids, eicosanoids

## Abstract

**Background:**

Analyses of brain samples from Alzheimer’s disease (AD) patients may be expected to help us improve our understanding of the pathogenesis of AD. Bioactive lipids, including sphingolipids, glycerophospholipids, and eicosanoids/related mediators have been demonstrated to exert potent physiological actions and to be involved in the pathogenesis of various human diseases. In this cross-sectional study, we attempted to elucidate the associations of these bioactive lipids with the pathogenesis/pathology of AD through postmortem studies of human brains.

**Methods:**

We measured the levels of glycerophospholipids, sphingolipids, and eicosanoids/related mediators in the brains of patients with AD (AD brains), patients with Cerad score B (Cerad-b brains), and control subjects (control brains), using a liquid chromatography-mass spectrometry method; we also measured the mRNA levels of specific receptors for these bioactive lipids in the same brain specimens.

**Results:**

The levels of several species of sphingomyelins and ceramides were higher in the Cerad-b and AD brains. Levels of several species of lysophosphatidic acids (LPAs), lysophosphatidylcholine, lysophosphatidylserine, lysophosphatidylethanolamine (LPE), lysophosphatidylinositol, phosphatidylcholine, phosphatidylserine (PS), phosphatidylethanolamine (PE), phosphatidylinositol, and phosphatidylglycerol were especially high in the Cerad-b brains, while those of lysophosphatidylglycerol (LPG) were especially high in the AD brains. Several eicosanoids, including metabolites of prostaglandin E2, oxylipins, metabolites of epoxide, and metabolites of DHA and EPA, such as resolvins, were also modulated in the AD brains. Among the lipid mediators, the levels of S1P2, S1P5, LPA1, LPA2, LPA6, P2Y10, GPR174, EP1, DP1, DP2, IP, FP, and TXA2r were lower in the AD and/or Cerad-b brains. The brain levels of ceramides, LPC, LPI, PE, and PS showed strong positive correlations with the Aβ contents, while those of LPG showed rather strong positive correlations with the presence of senile plaques and neurofibrillary tangles. A discriminant analysis revealed that LPG is especially important for AD and the LPE/PE axis is important for Cerad-b.

**Conclusions:**

Comprehensive lipidomics, together with the measurement of lipid receptor expression levels provided novel evidence for the associations of bioactive lipids with AD, which is expected to facilitate future translational research and reverse translational research.

## Introduction

Alzheimer’s disease (AD) is the most prevalent form of dementia worldwide, and there still remain substantial unmet needs for the treatment of AD. Therefore, identification of treatment targets for AD, especially using human autopsy brain samples, is desired. Since apolipoprotein E (ApoE) polymorphism has been shown as a major risk factor for AD (Corder et al., 1993; Saunders et al., 1993) and ApoE-rich HDL is the major lipoprotein in the central nervous system (CNS; Vance and Hayashi, 2010), the involvement of lipids in the pathogenesis of AD has been investigated. Until date, several studies have been conducted to elucidate the modulations in the levels of various lipids and their involvement in the pathogenesis of AD (Yin, 2022). A series of elegant basic studies has demonstrated that bioactive lipids, such as sphingolipids, glycerophospholipids, and eicosanoids/related mediators, which are derived from polyunsaturated fatty acids, exert potent physiological actions and are involved in the pathogenesis of many diseases in humans (Cartier and Hla, 2019; Green et al., 2021; Dyall et al., 2022; Kano et al., 2022).

The brain is rich in sphingolipids, and several studies have investigated the modulations of sphingolipid levels in AD brains. Among the sphingolipids, the biological properties of sphingosine 1-phoshate (S1P) and ceramides have been the best studied in the field of neurology (De La Monte, 2012; Ghasemi et al., 2016). S1P is produced from sphingosine (Sph) by S1P kinases (Maceyka et al., 2002), and at present, five kinds of S1P receptors, S1P1–S1P5, have been identified. Ceramides are derived from sphingomyelins (SMs) and can be converted into Sph. In addition to S1P, dihydrosphingosine 1-phosphate (dhS1P) is also known as another ligand for S1P receptors. DhS1P is produced from dihydrosphingosine (dhSph), and dhSph can also be processed into ceramides *via* dihydroceramides (Bartke and Hannun, 2009). Elevated levels of SMs (Varma et al., 2018) and decreased levels of S1P (He et al., 2010) have been reported in AD brains. In regard to the ceramides, reports are controversial; some studies have reported increased ceramide levels (Satoi et al., 2005; Filippov et al., 2012; Varma et al., 2018), while one study has reported decreased ceramide levels in AD brains (Barbash et al., 2017). One study suggested that the modulations of the sphingolipid levels in the brain might depend on the type of AD (Cutler et al., 2004). Moreover, the modulations in the Sph and dhSph levels and the expression patterns of S1P receptors in AD brains remain uncertain.

Among the glycerophospholipids, lysophosphatidic acids (LPAs), which are produced from lysophosphatidylcholine (LPC) by autotaxin, are well studied (Kano et al., 2022). The levels of LPAs in the cerebrospinal fluid (CSF) are reported to be positively associated with the presence/absence of AD (Ahmad et al., 2020). Basic studies have revealed the involvement of LPAs in the development of CNS, in neuroplasticity, and in glial activation; LPAs have also been shown to play important roles in the accumulation of amyloid b protein (Ab) in the brain (Hao et al., 2020). However, strong evidence for the modulations of LPA and LPA receptor expression levels in human brains is still lacking. Moreover, modulations of the levels of LPC, a precursor of LPAs, and of phosphatidylcholine (PC), a precursor of LPC, are not yet well known. PC levels have been reported to be depressed in AD brains (Nitsch et al., 1992; Varma et al., 2018), while modulations of the LPA and LPC levels in AD brains remain unknown. In addition to the receptors for LPAs, three types of specific receptors for lysophosphatidylserine (LPS), namely GPR34, P2Y10, and GPR174 (Inoue et al., 2012), and a specific receptor for lysophosphatidylinositol (LPI) and lysophosphatidylglycerol (LPG), namely GPR55 (Oka et al., 2007), have also been identified. The glycero-lysophospholipid mediators also exert potent bioactivities through their receptors (Makide et al., 2014). LPS is produced from phosphatidylserine (PS), and LPI and LPG are produced from phosphatidylinositol (PI) and phosphatidylglycerol (PG), respectively. Until, the modulations of these lipids and of their receptor expressions have not yet been investigated in human AD brains. Although no specific receptor for this lipid molecule has been identified, the levels of lysophosphatidylethanolamine (LPE) are also dynamically modulated in several human diseases (Emoto et al., 2017; Kurano et al., 2017). Modulations of the LPE levels in human AD brains remain unknown, whereas the levels of phosphatidylethanolamine (PE), its precursor, have been reported to be depressed in AD brains (Nitsch et al., 1992).

In regard to modulations of the levels of fatty acids and their derivatives in AD brains, decrease in the DHA content in AD brains is almost well established (Astarita et al., 2010; Martin et al., 2010; Snowden et al., 2017), and the contents of other fatty acids are also reported to be decreased (Snowden et al., 2017). The metabolites derived from arachidonic acid (AA), the so-called eicosanoids, are known to be involved in the process of inflammation. The levels of prostaglandin (PG) E2, PGD2, PGF2a, and thromboxane (TX) B2 are reported to be elevated in AD brains (Iwamoto et al., 1989; Wong et al., 1992; Prasad et al., 1998). Although reports are still scarce at present, levels of metabolites of fatty acids other than AA may also be modulated in AD brains. The levels of DHA derivatives, such as maresin 1 and resolvin D5, are reported to be lower in AD brains (Zhu et al., 2016). Levels of anandamide (AEA), an endocannabinoid, are reported to be decreased in AD brains (Jung et al., 2012). However, there is still insufficient evidence of the modulations of the levels of eicosanoids and related metabolites in AD brains, as no comprehensive measurements of these numerous metabolites have been performed yet. Moreover, studies of modulations in the expressions of specific receptors of the eicosanoids, such as DP1, DP2, EP1-4, FP, IP, and TXAr2 are also warranted, since they are of interest as pharmacotherapeutic targets.

Considering this background, together with the recent advances in lipidomics using the liquid chromatography-mass spectrometry (LC–MS/MS) system, which can be applied for precise measurements of the levels of lipid metabolites in human samples, we simultaneously investigated the modulations in the levels of glycero-lysophospholipids, diacyl-phospholipids, sphingolipids, and eicosanoids/related mediators, as well as those of the mRNA levels of specific receptors for these lipid mediators, in the postmortem brains of AD patients, patients with Cerad score B (Cerad-b), intermediate probability of AD (Mirra et al., 1991), and control subjects, in order to elucidate the involvement of theses bioactive lipids in the pathogenesis of AD and facilitate future translational research to develop novel treatments.

## Materials and methods

### Samples

We conducted this study using 19 autopsied brain specimens, obtained from 6 AD patients, 7 Cerad-b patients, and 6 normal control subjects who showed no evidence of other CNS disorders in Tokyo Metropolitan Geriatric Medical Center. Frozen postmortem cerebral cortex specimens were used for the measurements. Cerad score is a semiquantitative measure of neuritic plaques and Cerad-b is classified as intermediate probability of AD (Murayama and Saito, 2004). All the autopsied specimens were obtained from the Brain Bank or Aging Research. The clinical phenotypes, including the diagnostic group (control, Cerad-b, or AD), grades of senile plaque (SPs), and Braak stage were evaluated based on histopathological examination of the specimens. The Ab contents were measured using a Human β Amyloid (1–42) ELISA kit (298–62,401, WAKO Pure Chemical Industries, Osaka, Japan), and adjusted to the brain protein levels. The characteristics of the subjects, as well as ApoE genotypes, are described in Table 1.

**Table 1:** Characteristics of the subjects.

AD	Age	Sex	SP	Braak	Ab	ApoE
a1	86	F	3	5	1.342	3/4
a2	87	F	3	5	1.515	3/4
a3	85	M	3	5	1.258	2/3
a4	88	M	3	5	1.904	3/4
a5	86	F	3	5	1.676	3/3
a6	85	M	3	5	1.851	3/4
Average	86.17 ± 1.07	M/*F* = 3/3	3.00 ± 0.00	5.00 ± 0.00	1.591 ± 0.242	
Cerad-b						
b1	86	F	2	2	2.048	3/3
b2	89	F	1	2	1.757	3/3
b3	81	M	2	2	1.207	3/4
b4	89	F	2	2	1.243	3/3
b5	86	F	1	1	1.231	3/3
b6	87	M	1	1	2.172	3/3
b7	80	F	2	1	1.396	3/4
Average	85.43 ± 3.33	M/*F* = 2/5	1.57 ± 0.49	1.57 ± 0.49	1.579 ± 0.380	
Control						
c1	89	M	1	1	0.653	3/3
c2	81	M	1	1	0.536	3/3
c3	86	F	1	2	1.001	3/3
c4	83	M	0	1	0.493	3/3
c5	80	F	0	2	0.595	3/3
c6	80	M	1	1	0.539	3/3
Average	83.17 ± 3.34	M/*F* = 4/2	0.67 ± 0.47	1.33 ± 0.47	0.636 ± 0.171	

The current cross-sectional study was performed in accordance with the ethical guidelines laid down in the Declaration of Helsinki. Written informed consent was obtained in advance from the brain donors and/or the next of kin. The study design was approved by Tokyo Metropolitan Geriatric Medical Center and The University of Tokyo Medical Research Center Ethics Committee (2018088NI).

### Measurement of the levels of S1P, ceramides and sphingosine, glycero-lysophospholipids, diacyl-phospholipids, and eicosanoids/related mediators

We measured the levels of the lipid mediators listed below by five independent LC–MS/MS methods using an LC8060 system, consisting of a quantum ultra-triple quadrupole mass spectrometer (Shimadzu, Japan). We simultaneously measured six ceramide species (Cer d18:1/16:0 [C16:0], Cer d18:1/18:0 [C18:0], Cer d18:1/18:1 [C18:1], Cer d18:1/20:0 [C20:0], Cer d18:1/22:0 [C22:0], Cer d18:1/24:0 [C24:0]), Sph, and dhSph, as previously described (Morita et al., 2020). We also measured the levels of S1P and dhS1P as described previously (Sakai et al., 2020). Furthermore, the levels of LPA, LPC, LPS, LPI, LPG, and LPE were also measured, as described previously (Kurano et al., 2015a,b; Morita et al., 2019). In the present study, we monitored 11 acyl chains (14: 0, 16:0, 16:1, 18:0, 18:1, 18:2, 18:3, 20:3, 20:4, 20:5, and 22:6) for these lysophospholipids and 22:5 LPI. We also measured the levels of SM, PC, PE, PG, PI, and PS (Kurano et al., 2019, 2022). We monitored 17 diacyl chains for SM and 64 diacyl chains for PC, PE, PI, PG, and PS. We also measured the levels of 193 eicosanoids/related mediators, together with 18 internal standards, AA, EPA, and DHA, as described previously (Morita et al., 2021). In all of these measurements, except those of SM and diacyl-phospholipids, both the intra-day and inter-day coefficients of variation are below 20%, as validated in our previous studies. The lipid contents were adjusted to the protein levels.

### Reverse-transcriptase PCR

Total RNA extracted from murine tissues or cells using the GenElute Mammalian Total RNA Miniprep kit was subjected to reverse transcription with the ReverTra Ace qPCR RT Master Mix. Quantitative PCR was performed using an ABI 7300 Real-Time PCR System (Applied Biosystems), using the primers for S1P and several LPA receptors described in previous reports (Enooku et al., 2016; Uranbileg et al., 2018) and commercially available primers listed in Supplementary Table S1. The expression levels of the genes of interest were normalized to those of the endogenous control *18 s* mRNA.

### Statistical analysis

**Figure 1:**
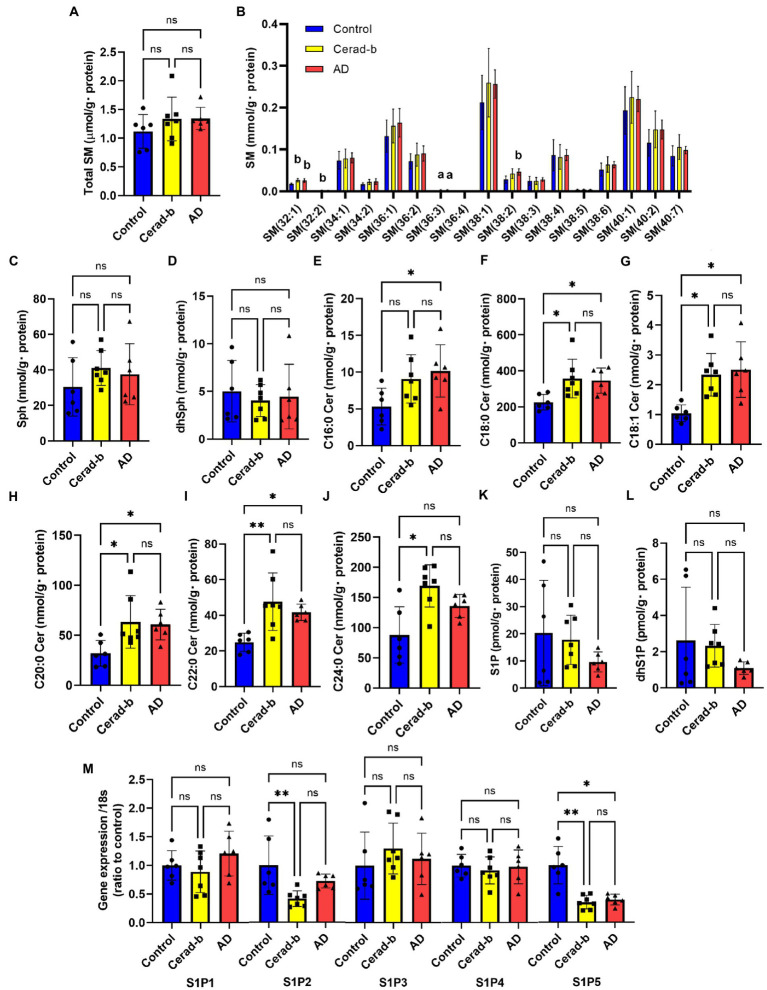
Modulations of sphingolipid levels and mRNA levels of S1P receptors in the brain of AD and Cerad-b. The sphingolipid levels and the mRNA levels of S1P receptors were measured in the brain of control subjects (*n* = 6), the patients with Cerad-b (*n* = 7), and those with AD (*n* = 6). The Kruskal-Wallis test, followed by the Steel-Dwass test as a post-hoc test, was used for statistical evaluation of the differences. **(A)** Total SM levels. **(B)** The brain levels of SM species. **(C)** Sph levels. **(D)** DhSph levels. **(E–J)** The brain ceramide levels. **(K)** S1P levels. **(L)** DhS1P levels. **(M)** The mRNA expression levels of S1P receptors adjusted to the expression level of 18 s as the internal standard. *p < 0.05; **p < 0.01; ns, not significant; a, *p* < 0.05 vs. control; b, *p* < 0.01 vs. control; c, *p* < 0.001 vs. control; d, *p* < 0.05 between Cerad-b and AD; e, *p* < 0.01 between Cerad-b and AD; f, *p* < 0.001 between Cerad-b and AD.

**Figure 2:**
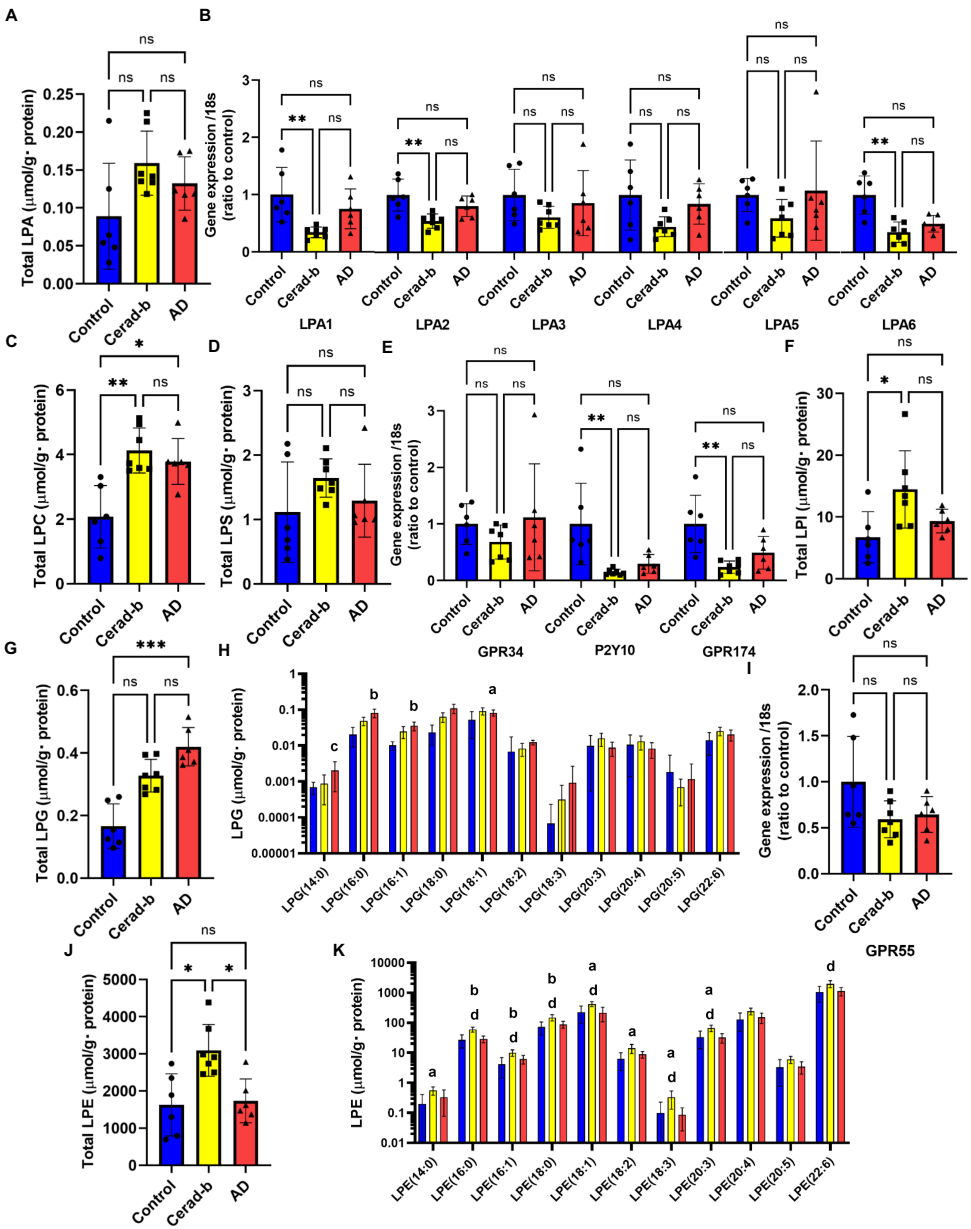
Modulations of the glycero-lysophospholipid levels and mRNA expression levels of LPA, LPS, and LPI/LPG receptors in the brain in AD and Cerad-b. The glycero-lysophospholipid levels and mRNA expression levels of LPA, LPS, and LPI/LPG receptors were measured in the brains of control subjects (*n* = 6), patients with Cerad-b (*n* = 7), and patients with AD (*n* = 6). The Kruskal-Wallis test, followed by the Steel-Dwass test as a post-hoc test, was used for statistical evaluation of the differences. **(A)** Total LPA levels. **(B)** The mRNA levels of the LPA receptors adjusted to the expression level of 18 s as the internal standard. **(C)** Total LPC levels. **(D)** Total LPS levels. **(E)** mRNA expression levels of LPS receptors adjusted to the expression level of 18 s as the internal standard. **(F)** Total LPI levels. **(G)** Total LPG levels. **(H)** LPG species. **(I)** mRNA levels of the LPI/LPG receptor, GPR55, adjusted to the expression level of 18 s as the internal standard. **(J)** Total LPE levels. **(K)** LPE species. *p < 0.05; **p < 0.01; ***p  < 0.001; ns, not significant; a, *p* < 0.05 vs. control; b, *p* < 0.01 vs. control; c, *p* < 0.001 vs. control; d, *p* < 0.05 between Cerad-b and AD; e, *p* < 0.01 between Cerad-b and AD; f, *p* < 0.001 between Cerad-b and AD.

**Figure 3:**
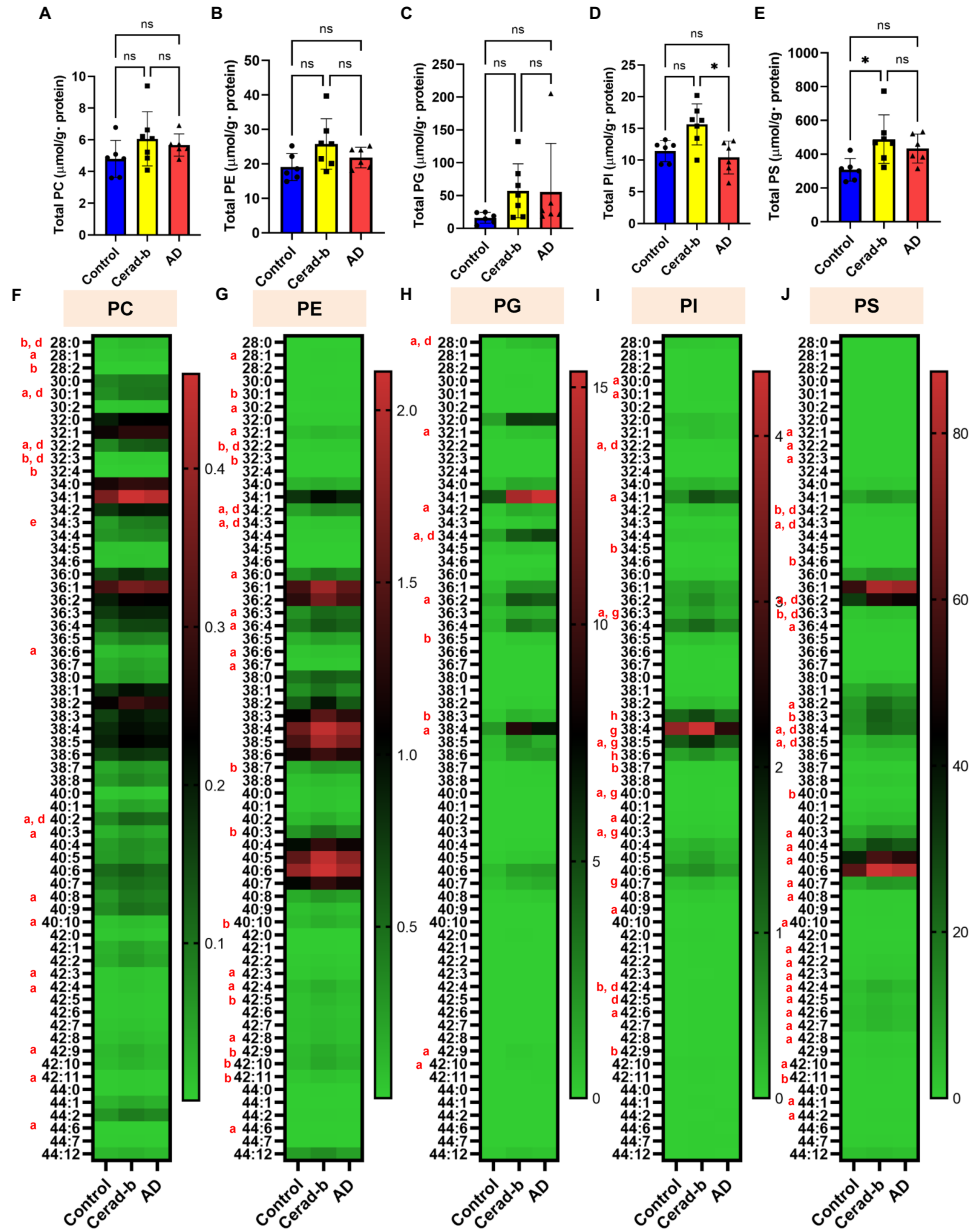
Modulations of the diacylphospholipid levels in the brains in AD and Cerad-b. The diacylphospholipid levels were measured in the brains of control subjects (*n* = 6), patients with Cerad-b (*n* = 7), and patients with AD (*n* = 6). The Kruskal-Wallis test, followed by the Steel-Dwass test as a *post hoc* test, was used to evaluate the difference. **(A)** Total PC levels. **(B)** Total PE levels. **(C)** Total PG levels. **(D)** Total PI levels. **(E)** Total PS levels. *p < 0.05. **(F)** PC species. **(G)** PE species. **(H)** PG species. **(I)** PI species. **(J)** PS species. ns, not significant; a, *p* < 0.05 Cerad-b vs. control; b, *p* < 0.01 Cerad-b vs. control; c, *p* < 0.001 Cerad-b vs. control; d, *p* < 0.05 AD vs. control; e, *p* < 0.01 vs. AD vs. control f, *p* < 0.001 AD vs. control; g, *p* < 0.05 Cerad-b vs. AD; h, *p* < 0.01 Cerad-b vs. AD; i, *p* < 0.001 Cerad-b vs. AD.

**Figure 4:**
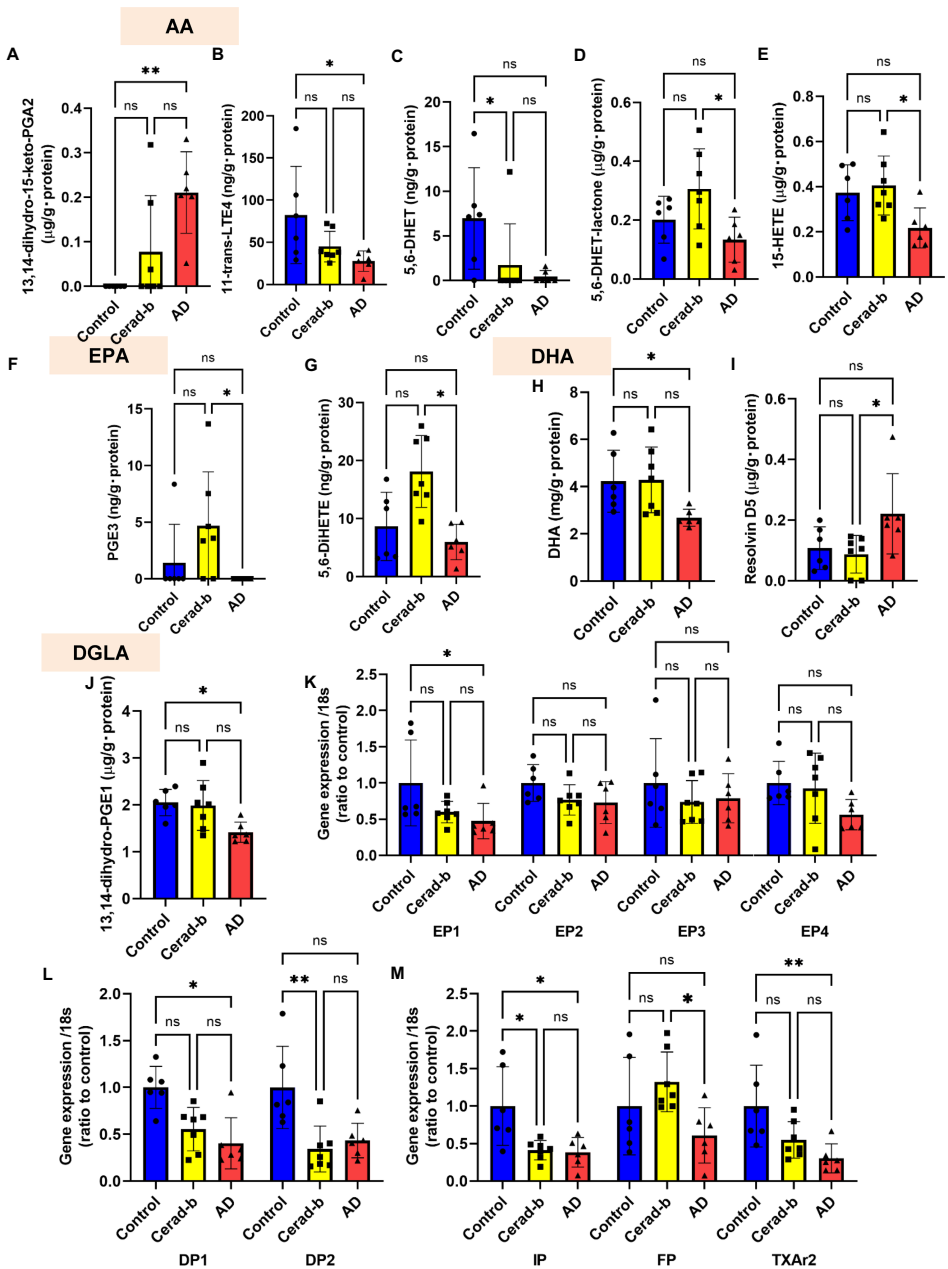
Modulations of the levels of eicosanoids and related mediators, and the eicosanoid receptor mRNA levels in the brains in AD and Cerad-b. The levels of eicosanoids and related mediators and the mRNA expression levels of the eicosanoid receptors were measured in the brains of control subjects (*n* = 6), patients with Cerad-b (*n* = 7), and patients with AD (*n* = 6). The Kruskal-Wallis test, followed by the Steel-Dwass test as a post-hoc test, was used for statistical evaluations of the differences. **(A)** 13,14-dihydro-15-keto PGA2 levels. **(B)** 11-trans-LTE4 levels. **(C)** 5,6-DHET levels. **(D)** 5,6-DHET-lactone levels. **(E)** 15-HETE levels. **(F)** PGE3 levels. **(G)** 5,6-DiHETE levels. **(H)** DHA levels. **(I)** Resolvin D5 levels. **(J)** 13,14-dihydro-PGE1 levels. **(K–M)** The mRNA levels of the eicosanoid receptor, GPR55, adjusted to that of 18 s as the internal standard. *p < 0.05; **p < 0.01; ns, not significant.

**Figure 5:**
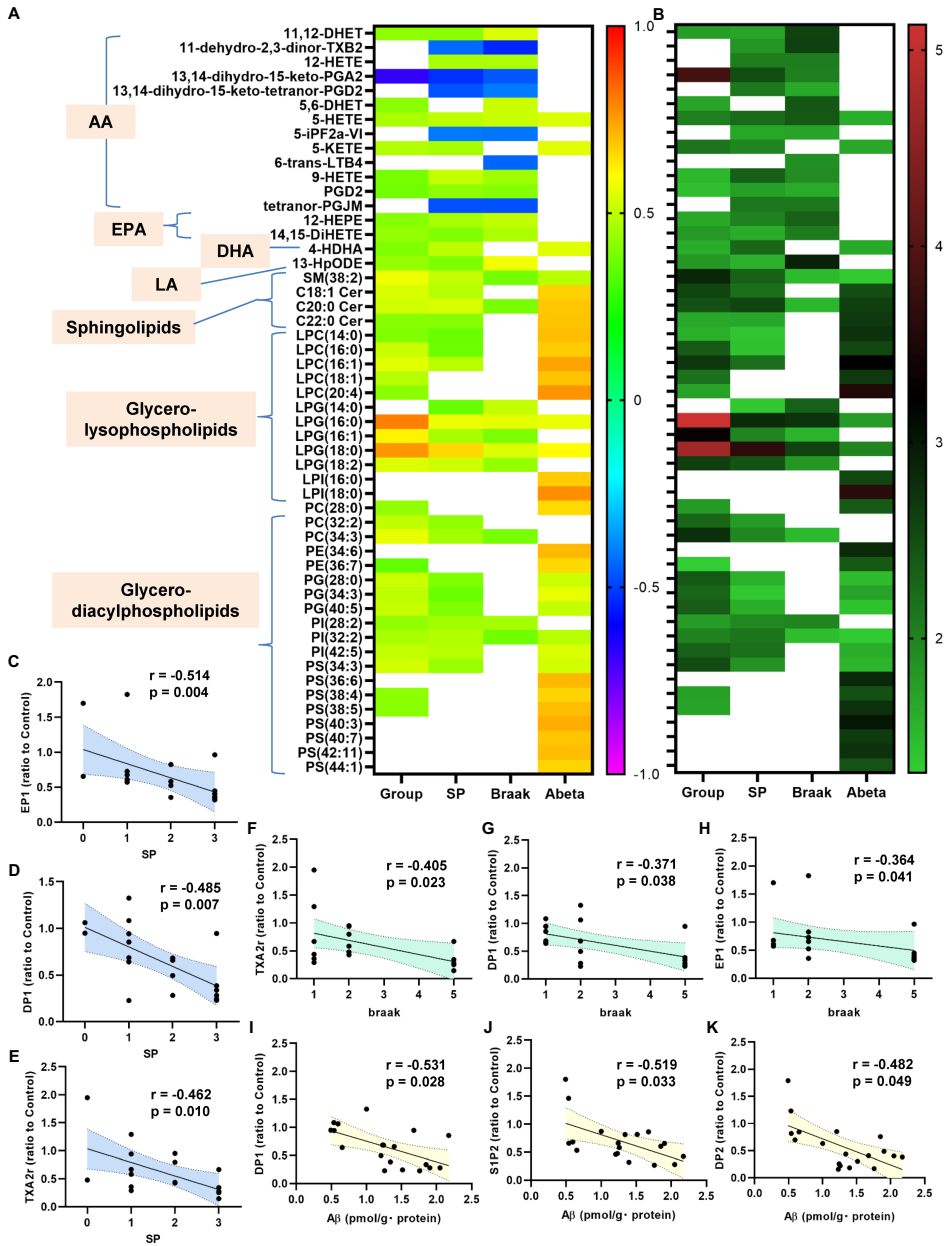
Correlations between bioactive lipids/their receptors and the clinical phenotypes. The correlations between the bioactive lipid/their receptor expression levels and the clinical phenotypes (group [control, Cerad-b, and AD], SP, Braak stage, and Ab) were examined using the Kendall rank correlation for correlations with the diagnostic group, SP, and Braak stage, and the Spearman rank correlation for Ab, using age and sex as the covariates of interest. **(A,B)** The correlation coefficients and the *p*-values of the top 20 lipid mediators with the lowest *p* values in the analysis of the correlations with any specific clinical parameters (group [control, Cerad-b, and AD], SP, Braak stage, and Ab) as heat maps. **(C–K)** The significant correlations between the lipid receptor expressions and clinical phenotypes.

**Figure 6:**
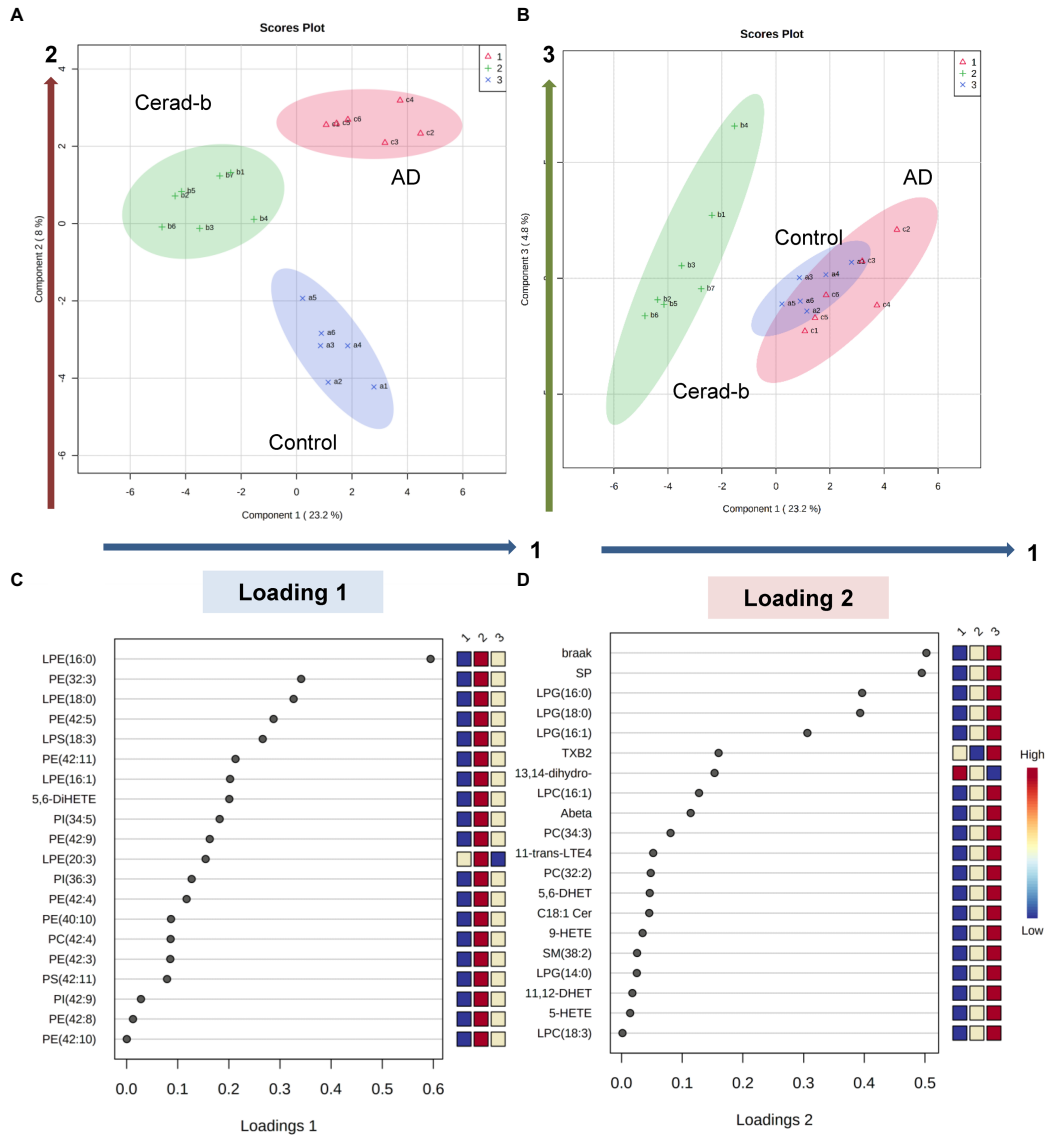
Discriminant analysis using the brain levels of bioactive lipids for diagnostic groups. Sparse PLS-DA was performed to identify bioactive lipids that contribute to discriminating AD from Cerad-b and control, and those that contribute to discriminating AD and Cerad-b from control. **(A,B)** Score plots. **(C)** Loading plot for component 1. **(D)** Loading plot for component 2.

The data were analyzed using SPSS (Chicago, IL) or MetaboAnalyst 5.0.^1^ To examine the statistical significances of differences in the lipid levels and mRNA expression levels of the lipid receptors among the control brains, Cerad-b brains, and AD brains, we used the Kruskal-Wallis test, followed by the Steel-Dwass test as a post-hoc test (Figures 1–4). For the correlation studies, the Kendall rank correlation was used to examine the associations of the levels of the lipids and of the mRNA expression levels of the lipid receptors with the clinical phenotypes (diagnostic group, SP score, and Braak stage), and the Spearman rank correlation was used to examine the association of the lipids with the contents of Ab, considering age and sex as covariates of interest (Figure 5). A sparse OPLS-DA was performed by MetaboAnalyst using three components to which a maximum of 20 variables can contribute, with the results of the lipid levels together with the age, sex, SP score, Braak stage, and content of Ab, to explore the characteristics of the three diagnostic groups (Figure 6). The graphic figures were prepared using Graphpad Prism 9 (GraphPad Software, San Diego, CA) or MetaboAnalyst. *p* values of less than 0.05 were deemed as denoting statistical significance in all the analyses.

## Results

### The levels of several species of sphingomyelin and ceramides were higher in the Cerad-b and Alzheimer’s disease brains

Figure 1 shows the modulations in the levels of sphingolipids and S1P receptors in the Cerad-b and AD brains. Although the total SM levels were not modulated, the SM (32:1), SM (32:2), SM (36:3), and SM (38:3) levels were higher in the Cerad-b and/or AD brains (Figures 1A,B). The Sph and dhSph levels were not significantly different (Figures 1C,D). The brain levels of ceramides were higher in the Cerad-b and/or AD brains (Figures 1E–J). The S1P and dhS1P levels tended to be lower in the AD brains, although the difference did not reach statistical significance (Figures 1K,L). In regard to the mRNA expression levels of the S1P receptors, the mRNA levels of S1P2 and S1P5 were lower in the Cerad-b brains, and those of S1P5 were lower in the AD brains (Figure 1M).

### The levels of several species of lysophosphatidic acid, lysophosphatidylcholine, lysophosphatidylserine, lysophosphatidylethanolamine, and lysophosphatidylinositol were higher especially in the Cerad-b brains, while those of lysophosphatidylglycerol were higher especially in the Alzheimer’s disease brains

Figure 2 shows the modulations of glycero-phospholipids and their receptors in the Cerad-b and AD brains. Although the total LPA levels were not significantly modulated, the LPA (18:0) levels were higher in the Cerad-b brains (Figure 2A; Supplementary Figure S1A). Among the LPA receptors, the mRNA levels of LPA1, LPA2, and LPA6 were lower in the Cerad-b brains (Figure 2B). The levels of LPC, a precursor of LPAs, were generally higher in the Cerad-b and AD brains (Figure 2C; Supplementary Figure S1B), although the mRNA levels of autotaxin, an enzyme catalyzing the production of LPAs from LPC, were not significantly modulated (Supplementary Figure S2). In regard to LPS, although the total LPS levels were not significantly modulated, the LPS (18:0) and LPS (18:3) levels were higher in the Cerad-b brains (Figure 2D and Supplementary Figure S1C). Among the LPS receptors, the mRNA levels of P2Y10 and GPR174 were lower in the Cerad-b brains, and tended to be lower in the AD brains (Figure 2E). The brain levels of LPI, especially of LPI (16:0) and LPI (18:0), were higher in the Cerad-b brains, while those of LPG were higher especially in the AD brains (Figures 2F–H; Supplementary Figure S1D). The mRNA levels of GPR55, a receptor for LPI and LPG, were not significantly modulated (Figure 2I). The brain levels of LPE were generally higher in the Cerad-b brains (Figures 2J,K).

### The levels of several species of phosphatidylcholine, phosphatidylserine, phosphatidylinositol, phosphatidylglycerol, and phosphatidylethanolamine were higher in the Cerad-b and Alzheimer’s disease brains

Figure 3 shows the modulations of diacylglycerols in the Cerad-b and AD brains. The total levels of PC, PE, and PG were not different, while those of several PC, PE, and PG species were positively modulated in the Cerad-b and AD brains, especially in the Cerad-b brains (Figures 3A–C,F). The total levels of PI, as well as those of several species of PI, were higher in the Cerad-b brains than in the AD brains (Figures 3D,I). The levels of several PI species were higher in the Cerad-b brains than in the control brains. The total levels of PS, as well as the levels of several species of PS were higher in the Cerad-b brains than in the control brains (Figures 3E,J). The levels of several PS species were also higher in the AD brains than in the control brains.

### Several classes of eicosanoids/related mediators were modulated in the Cerad-b and Alzheimer’s disease brains

Figure 4 shows the significant modulations of eicosanoids and related metabolites, and also those of their specific receptors in the Cerad-b and AD brains. Among the eicosanoids, the brain levels of 13,14-dihydro-15-keto PGA2, a PGE2-derived metabolite, were higher (Figure 4A), while those of 11-trans-LTE4, an LTC4 metabolite, were lower (Figure 4B) in the AD brains. The levels of 5,6-DHET, an AA-derived epoxide metabolite, were lower in the Cerad-b brains and and tended to be lower in the AD brains, and those of 5,6-DHET-lactone, a metabolite of 5,6-DHET, were lower in the AD brains than in the Cerad-b brains (Figures 4C,D). The levels of 15-HETE, an oxylipin, were also lower in the AD brains than in the Cerad-b brains (Figure 4E). Among the EPA-derived metabolites, the levels of PGE3 and 5,6-DiHETE were lower in the AD brains than in the Cerad-b brains (Figures 4F,G). The levels of DHA were lower in the AD brains than in the control brains and those of resolvin D5 were lower in the AD brains than in the Cerad-b brains (Figures 4H,I). In regard to the metabolites of other fatty acids, the levels of 13,14-dihydro-PGE1, a metabolite of PGE1, which is produced from dihomo-γ-linoleic acid, were lower in the AD brains (Figure 4J). Among the specific receptors for eicosanoids, the mRNA levels of PGE2 receptor 1 (EP1), PGD2 receptor 1 (DP1), PGI2 receptor (IP), and TXA2 receptor (TXA2r) were lower in the AD brains, while those of DP2 and IP were lower in the Cerad-b brains (Figures 4K–M). The mRNA levels of the PGF receptor (FP) were lower in the AD brains than in the Cerad-b brains (Figure 4M).

### Correlations of the levels of bioactive lipids and of their receptor expression levels with the clinical phenotypes as determined by brain autopsy

Next, we investigated the correlations of the levels of bioactive lipids with the clinical phenotypes. Figures 5A,B shows the correlation coefficients and the *p*-values of the top 20 lipid mediators with the lowest *p* values in the analysis of the correlations with any specific clinical parameters (group [control, Cerad-b, and AD], SP score, Braak stage, and Ab content) as heat maps. Among the metabolites of AA, the metabolites which showed positive correlations with clinical phenotypes were 11,12-DHET and 5, 6-DHET (AA-derived epoxide metabolites), 12-HETE, 5-HETE, 5-KETE, and 9-HETE (oxylipins produced by LOX), and PGD2, while the metabolites which showed negative correlations with the clinical phenotypes were 11-dehydro-2,3-dinor-TXB2 (a TXA2 metabolite), 13,14-dihydro-15-keto PGA2 (a PGE2-derived metabolite), 5-iPF2a-VI (an isoprostane), and tetranor-PGJM (a PGD2 metabolite). The levels of 12-HEPE and 14,15-DiHETE (EPA metabolites) and 4-HDHA (a DHA metabolite), and 13-HpODE (a linoleic acid metabolite) also showed positive correlations with the clinical phenotypes.

Among the sphingolipids, SM (38:2), and the C18:1, C20:0, and C22:0 ceramides showed positive correlations with the clinical phenotypes, and we observed strong positive correlations especially between the levels of the ceramides and the Ab contents. In regard to the glycero-lysophospholipids, some species of LPC and LPI showed strong positive correlations with the Ab contents, while some LPG species showed strong positive correlations with the diagnostic group, SP score, and Braak stage. Among the diacylglycerols, some species of PC, PG, and PI showed rather strong positive correlations with the diagnostic group, SP score, and Braak stage, while some species of PE and PS showed positive strong correlations with the Ab contents.

In regard to the association of the expressions levels of the lipid receptors with the clinical parameters, the mRNA levels of TXAr2, DP1, EP1, IP, and S1P5 showed significantly negative correlations with the diagnostic group (Supplementary Figure S3). The mRNA levels of EP1, DP1, and TXAr2 were negatively correlated with the levels of SP, those of TXAr2, DP1, and EP1 were negatively correlated with the Braak stage, and those of DP1, S1P2, and DP2 were negatively correlated with the Ab contents (Figures 5C–K).

### Sparse PLS-DA identified bioactive lipids which contributed to discriminating Alzheimer’s disease brains from Cerad-b and control brains and those which contributed to discriminating Alzheimer’s disease and Cerad-b brains from control brains

Lastly, we performed a sparse PLS-DA to identify the bioactive lipids that contributed to discriminating among the diagnostic groups, considering the clinical phenotypes together. As shown in Figures 6A,B, component 1 allowed Cerad-b brains to be discriminated from the control and AD brains, whereas component 2 allowed discrimination of each group from the other diagnostic groups. Component 3 explained the individual differences among the groups. The levels of LPE (16:0), PE (32:3), LPE (18:0), PE (42:5), LPS (18:3), PE (42:11), LPE (16:1), and 5,6-DiHETE contributed to component 1 (Figure 6C). The Braak stage and SP score contributed most strongly to component 2, followed by the levels of LPG (16:0), LPG (18:0), and LPG (16:1) (Figure 6D). The individual dot plots of the contributing metabolites are described in Supplementary Figure S4.

## Discussion

**Figure 7:**
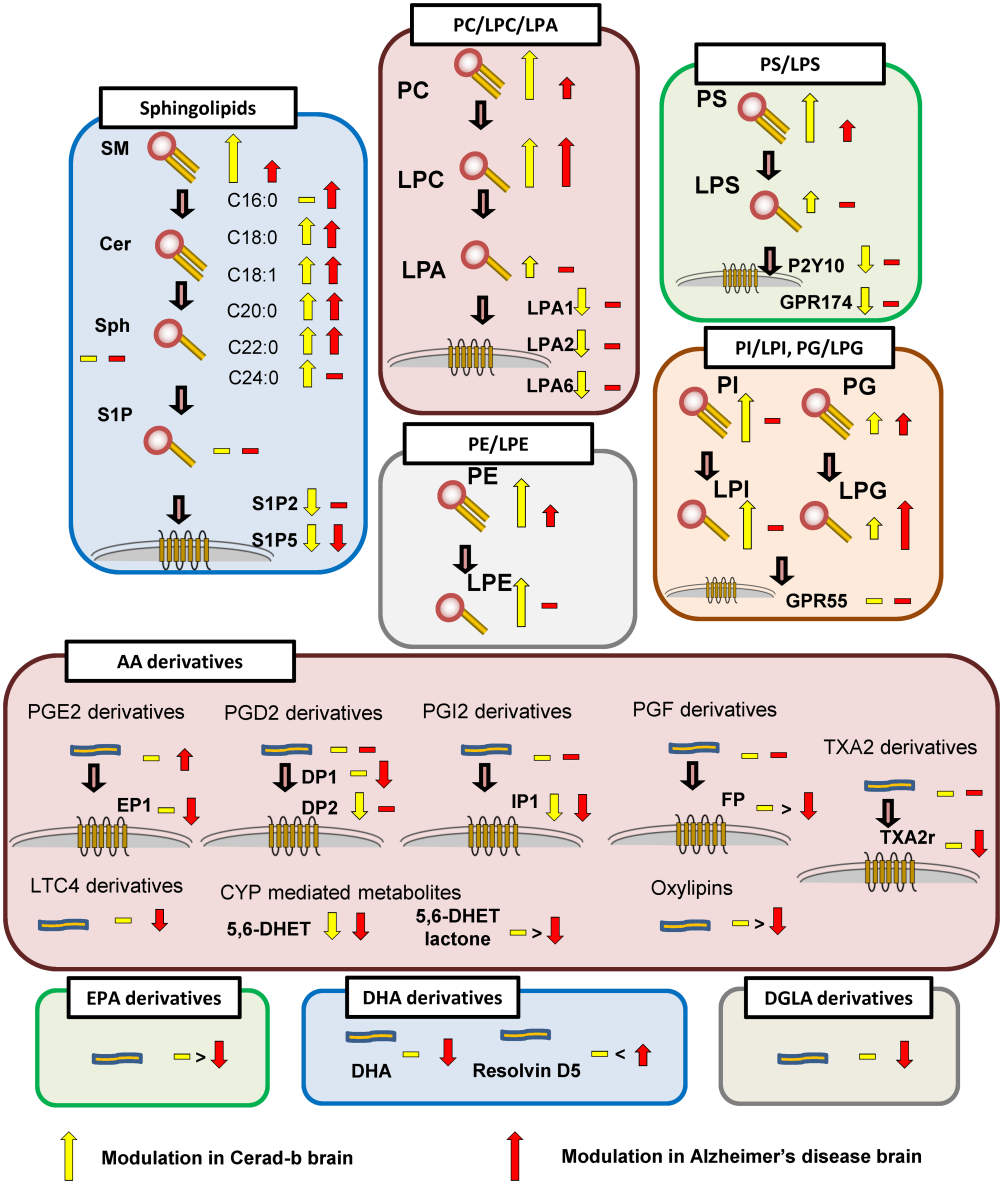
Schematic showing modulation of bioactive lipid species in the brain in AD.

In this study, we investigated the modulations of the levels of bioactive lipids, such as glycero-lysophospholipids, sphingolipids, and eicosanoids/related mediators, and of the mRNA expression levels of their receptors in Cerad-b and AD brains. The results are summarized in Figure 7.

Regarding the sphingolipids, the levels of several species of SM and ceramides were higher in the Cerad-b and AD brains, and were positively associated with the clinical phenotypes (Figures 1, 5). These results are consistent with several previous reports (Satoi et al., 2005; Filippov et al., 2012; Varma et al., 2018). Considering the bioactivities of ceramides, such as pro-apoptotic (Obeid et al., 1993) and pro-inflammatory (Vandanmagsar et al., 2011) activities, the elevated levels and positive correlations of the levels of ceramides with the clinical phenotypes seem reasonable. Although the brain contents of S1P were not significantly modulated in the present study, they tended to be lower in the AD brains, which is consistent with the results of a previous study (He et al., 2010). In regard to the responsible receptors, the mRNA levels of S1P2 and S1P5 were negatively modulated in the Cerad-b and/or AD brains. Although no involvement of S1P2 in the pathogenesis of AD has been reported, S1P5 is widely expressed in the CNS, and in one study, an S1P5 agonist was demonstrated to attenuate age-related cognitive decline in mice (Hobson et al., 2015). The present results suggest attenuated S1P5 signaling in the pathogenesis of AD, which might support the idea of the potential clinical usefulness of an S1P5 agonist in AD treatment in humans.

While only slight modulation of the levels of LPAs was observed, the mRNA levels of LPA1, LPA2, and LPA6 were decreased in the Cerad-b brains (Figures 2A,B; Supplementary Figure S1A). Regarding the associations between the expression levels of specific LPA receptors and AD, no direct associations have been reported, and only LPA1 has been reported to facilitate the brain delivery of donepezil (Choi et al., 2021); however, considering the important roles of LPA in the field of neurology, as described in the *Introduction* section (Hao et al., 2020), the present results suggest that the LPA receptors might be potential therapeutic targets for AD. The finding in this study that modulations of these LPA receptors were observed only in Cerad-b brains, and not AD brains, might be of some significance. We also observed elevation of the PC and LPC levels in the Cerad-b and AD brains (Figures 2C, 3A,F; Supplementary Figure S1B). In addition to the physiological significance as precursors of LPA (Uranbileg et al., 2021), LPC, in particular, possesses lipotoxicity (Chen et al., 2022; Yoshioka et al., 2022). Strong positive correlations of the levels of LPC and PC with the clinical phenotypes were observed (Figures 5A,B). The levels of several species of LPS/PS, LPE/PE, and LPI/PI were also higher in the Cerad-b brains (Figures 2D,F–H, 3B,D,E,G,I,J; Supplementary Figure S1C,D). Since the mRNA levels of P2Y10 and GPR174 were lower and those of GPR34 were not modulated in the Cerad-b brains (Figure 2E), the elevation of LPS in these brains might imply the specific activation of GPR34 signaling among the LPS receptors. Until date, no association of LPS with AD has been reported. Considering that GPR34 signaling activates the microglia (Sayo et al., 2019) and P2Y10 signaling suppresses inflammation of the microglia (Kita et al., 2019), these modulations observed in the Cerad-b brains may aggravate the pathogenesis of AD. In regard to PS, although involvement of the physiological actions of PS itself in the pathogenesis of AD remains unresolved, the intake of PS-containing foods has been reported to improve the phenotypes of AD (Zhang et al., 2015). In this study, the brain contents of PS showed strong positive correlations with the Ab contents. PS reportedly enhances the activity of BACE-1, an enzyme that catalyzes the production of Ab, which is consistent with the results of the present study (Kalvodova et al., 2005). In regard to LPE/PE, although the evidence might not be sufficient, suppression of PE generation has been reported to decreased Aβ accumulation (Nesic et al., 2012), suggesting that the modulations observed in the Cerad-b brains might promote the pathogenesis of AD, while one report demonstrated that LPE suppressed inflammatory changes and oxidative stress in microglial cells (Tsukahara et al., 2021), suggesting that elevation of LPE might be a compensatory change and that failure of this modulation might lead to the pathogenesis of AD from Cerad-b (Tsukahara et al., 2021). Regarding LPI/PI, while activation of GPR55, a receptor for LPI, has been reported to improve the pathological condition of AD (Xiang et al., 2022), a GPR55 antagonist has been reported to attenuate inflammatory changes of the microglia (Saliba et al., 2018), as of macrophages (Kurano et al., 2021). These axes are rather downregulated in AD brains as compared with the Cerad-b brains. To elucidate the significance of these modulations, basic researches are necessary to understanding the involvement in these lipids in the pathogenesis of AD with basic research is necessary. Compared with other glycerophospholipids, LPG/PG axis, especially LPG, is up regulated in AD and LPG had rather strong positive correlations with SP and Braak scores (Figures 2G,H, 3C,I, 5A,B). LPG seemed to characterize AD against control and Cerad-b (Figure 6D). This is the first study demonstrating the association between LPG and AD. LPG is another agonist for GPR55 (Oka et al., 2007). GPR55 is reportedly involved in a glia–neuron communication for neural development (Guy et al., 2015). Although the association has not been elucidated in neurology, LPGAT1, an enzyme involved in the metabolism of LPG/PG axis, has been demonstrated to be associated with mitochondria functions (Zhang et al., 2019). In addition, other biological properties are emerging now (Kawana et al., 2019). The further study on the involvement of LPG in the pathogenesis of AD, especially on the formation of SP, neurofibrillary tangle, and progression from Cerad-b to AD, is expected.

Compared with sphingolipids and glycerophospholipids, the contributions of eicosanoids and related lipid mediators to discriminate Cerad-b and/or AD seemed rather weak (Figure 6), however substantial changes were observed in the present study (Figures 4A–J). Regarding the modulations among three groups, the increase in a PGE2 metabolite and the decrease in DHA and its metabolite were concordant with the previous studies (Prasad et al., 1998; Astarita et al., 2010; Zhu et al., 2016). The decrease of 15-HETE in human brain of AD was firstly reported in this study, which is concordant with the previous study with mice (Tajima et al., 2013). The modulations of other mediators have not been reported previously. Regarding the physiological significance, since EP1 was down regulated in the brain of AD and PGE2 was not modulated (Figure 4K), the input of EP1 signal might be suppressed in the brain of AD. Reportedly, EP1 signal exacerbated neurotoxicity in murine brain (Zhen et al., 2012; Mendes et al., 2020), the down regulation of EP1 might be a compensatory modulation. The decrease of 11-trans-LTE4, a LTC4 metabolite, might also represent a compensatory change, considering their potent pro-inflammatory properties (Sasaki and Yokomizo, 2019), whereas the decrease of 15-HETE might accelerate the phenotypes of AD, since 15-HETE reportedly has anti-inflammatory properties (Profita et al., 2000). 5,6-DHET is an epoxide of AA, although its physiological properties are largely unknown at present. Among AA derivatives, it is interesting that several metabolites showed positive correlations, whereas others showed negative correlations with clinical phenotypes. The positive correlations of oxylipins produced by LOX or AA-derived epoxide metabolites might reflect the oxidative stress in the brain of Cerad-b or AD, while those of EPA or DHA metabolites might possess compensatory significances, considering their anti-inflammatory roles (Ishihara et al., 2019). 13-HpODE might be involved in the facilitating the pathogenesis of AD, considering its pro-inflammatory and pro-apoptotic properties (Dwarakanath et al., 2004; Ryman et al., 2016). 11-dehydro-2,3-dinor-TXB2 (a TXA2 metabolite) showed negative correlations and the mRNA levels of TXA2r were lower in the brain of AD and negatively correlated with clinical phenotypes. These results suggested the attenuation of TXA2 signal in AD. Considering that COX-2 has been proposed to possess deteriorating effects in AD pathophysiology (Tyagi et al., 2020), these associations might possess some significances, although the experimental research does not exist on these associations. Regarding other receptors for eicosanoids, the down regulation of DP1, DP2, IP, and FP was observed in the brain of Cerad-b and/or AD (Figures 4L,M). The result of DP1 was contrary to a previous report with a mice model of AD (Mohri et al., 2007) and the involvement of other receptors in AD has not been explored.

Since this is observational research with rather small number of subjects, understanding the detail mechanisms with basic studies and clinical studies is needed. However, comprehensive measurement of bioactive lipids in the brain of AD has not existed and we believe that this approach is important since these lipid mediators have the same origins, to some degree. For example, diacyl-phospholipids undergo hydrolysis into fatty acids, and lysophospholipids and eicosanoids and derivatives of w3 fatty acids can be produced from these fatty acids (Sato et al., 2016). Actually, the present study revealed that LPG is especially important for AD and LPE/PE axis is important only for Cerad-b (Figure 6).

In addition to the lipids we investigated in the present study, many other lipids exist in the brain and the lipids and their related proteins have been proposed to be associated with AD. As described in the *Introduction* section, the variant epsilon 4 of ApoE is established as a risk factor for AD (Corder et al., 1993). ApoE-rich HDL binds to LDL receptor and LRP1, which have been also demonstrated to be involved in the pathogenesis of AD (Shibata et al., 2000; Deane et al., 2004; Shi et al., 2021). Actually, in the samples we used in the present study, we observed the decrease of LRP1 in the brain of Cerad-b (Supplementary Figure S5). Moreover, cholesterol has been reported to be involved in the pathogenesis of AD (Rudajev and Novotny, 2022) and oxysterols such as 24S-hydroxycholesterol, a derivative of cholesterol (Mutemberezi et al., 2016), have been also demonstrated to have roles in the amyloidogenic process in AD (Sodero, 2021). Further investigation on the modulation of various lipids in the human postmortem samples would elucidate novel associations of lipids with AD in the future.

In summary, the present study revealed many novel potential associations between bioactive lipids or their receptors and AD in human brain. Especially, we could provide evidence for the involvement of ceramides in AD. We also elucidated the association of LPG/PG, LPE/PE, LPS/PS, and LPI/PI in human AD for the first time, which would facilitate future novel reverse translational research and also promote the translational research of elegant series of previous research in the fields of AD. The investigation on the modulations of lipid receptors will impact on the potential importance of S1P2, S1P5, LPA1, LPA2, LPA6, P2Y10, GPR174, EP1, DP1, DP2, IP, FP, and TXA2r as a target for AD.

## Data availability statement

The original contributions presented in the study are included in the article/Supplementary material, further inquiries can be directed to the corresponding author.

## Ethics statement

The studies involving human participants were reviewed and approved by Tokyo Metropolitan Geriatric Medical Center and the University of Tokyo Medical Research Center Ethics Committee. The patients/participants provided their written informed consent to participate in this study.

## Author contributions

MK designed the research. MK and BU analyzed the data. MK, YS, and BU performed the research. MK and YS administered the project. MK, YS, DS, KK, JA, and YY wrote the manuscript. All authors have read and approved the final manuscript.

## Funding

This work was supported by JSPS KAKENHI Grant Number 20H03573 (MK), the Takeda Science Foundation (MK), and Leading Advanced Projects for medical innovation (LEAP) from AMED.

## Conflict of interest

The authors declare that the research was conducted in the absence of any commercial or financial relationships that could be construed as a potential conflict of interest.

## Publisher’s note

All claims expressed in this article are solely those of the authors and do not necessarily represent those of their affiliated organizations, or those of the publisher, the editors and the reviewers. Any product that may be evaluated in this article, or claim that may be made by its manufacturer, is not guaranteed or endorsed by the publisher.

## Footnotes

^1^ http://www.metaboanalyst.ca/
