# Supplementary material for: Modulations of bioactive lipids and their receptors in postmortem Alzheimer’s disease brains
Source: Front Aging Neurosci. 2022 Dec 9;14:1066578. doi: 10.3389/fnagi.2022.1066578 (PMC9780287; doi:10.3389/fnagi.2022.1066578)
Supplement: Supplementary file 1 [file Presentation_1.PDF]

Supplemental Table S1. Probes used for real-time PCR in the present study

Gene name	Product number
LPA4	Hs00271072_s1
GPR34	Hs00271105_s1
P2Y10	Hs00274326_s1
GPR174	Hs00261404_s1
GPR55	Hs00271662_s1
EP1	Hs00909194_g1
EP2	Hs00168754_m1
EP3	Hs00168755_m1
EP4	Hs00168761_m1
DP1	Hs00235003_m1
DP2	Hs01867513_s1
IP	Hs01900573_s1
FP	Hs00168763_m1
TXA2r	Hs00169054_m1
LDL receptor	Hs01092524_m1
LRP1	Hs00233856_m1
ApoE	Hs00171168_m1
18s	Hs99999901_s1

Supplemental Figure S1

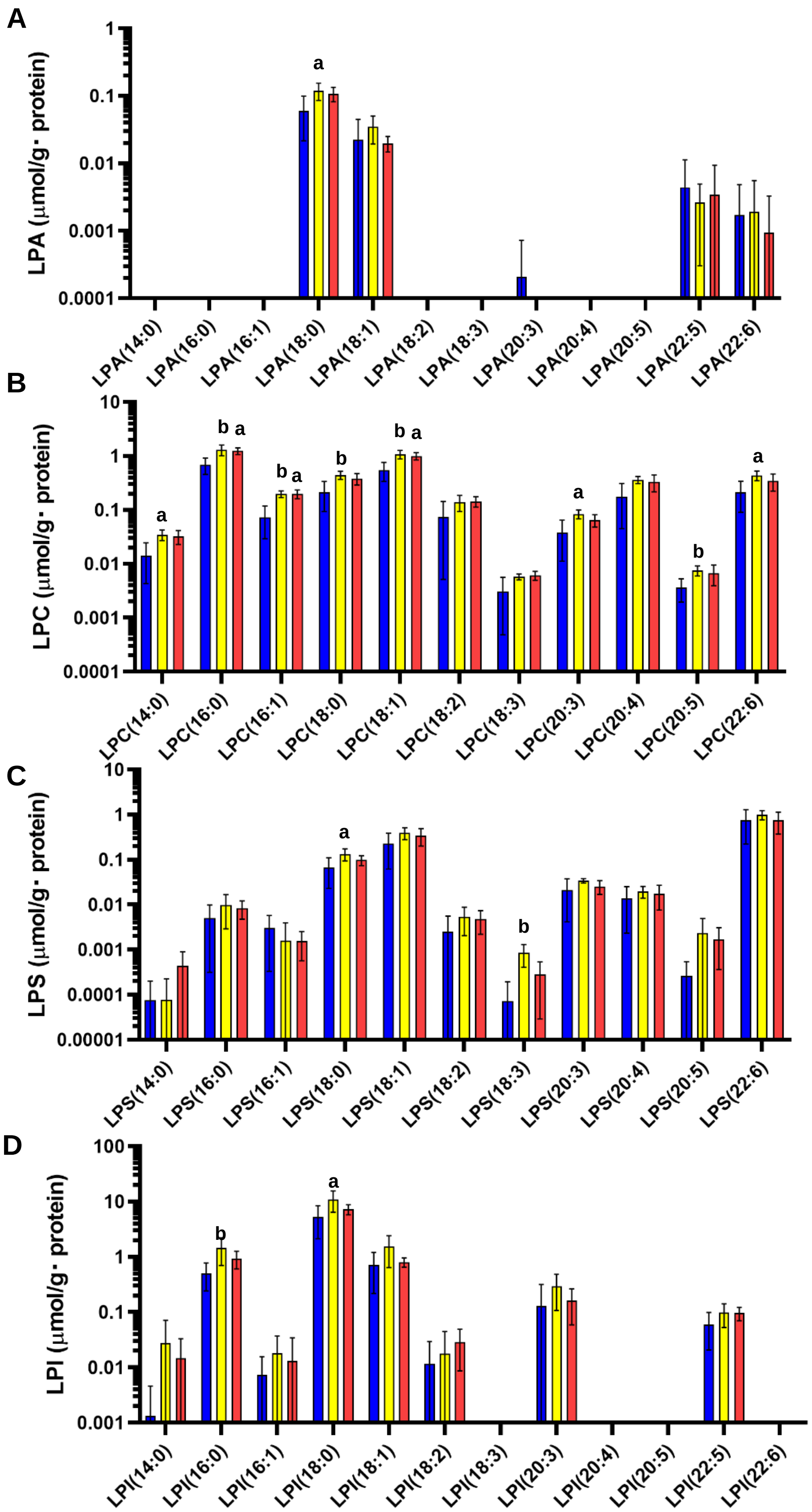

Supplemental Figure S1. Modulations of the LPA, LPC, LPI, and LPS species in the brain in AD and Cerad-b. The modulation of LPA species (A), LPC species (B), LPI species (C), and LPS species (D) in the analyses described in Figure 2 are shown. a: P<0.05 vs. control; b: P<0.01 vs. control; c: P < 0.001 vs. control; d: P<0.05 between Cerad-b and AD; e: P<0.01 between Cerad-b and AD; f: P<0.001 between Cerad-b and AD.

Supplemental Figure S2

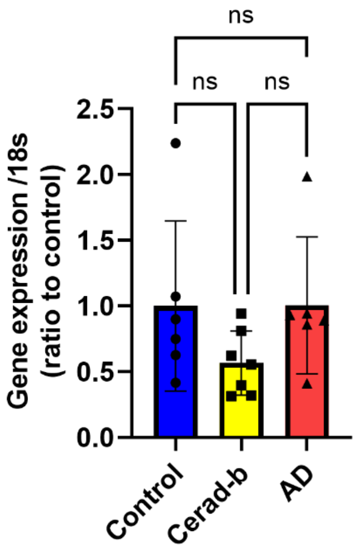

Supplemental Figure S2. Modulations of autotaxin mRNA levels in the brain of AD and Cerad-b. Modulation of the autotaxin mRNA levels in the analyses described in Figure 2.

Supplemental Figure S3

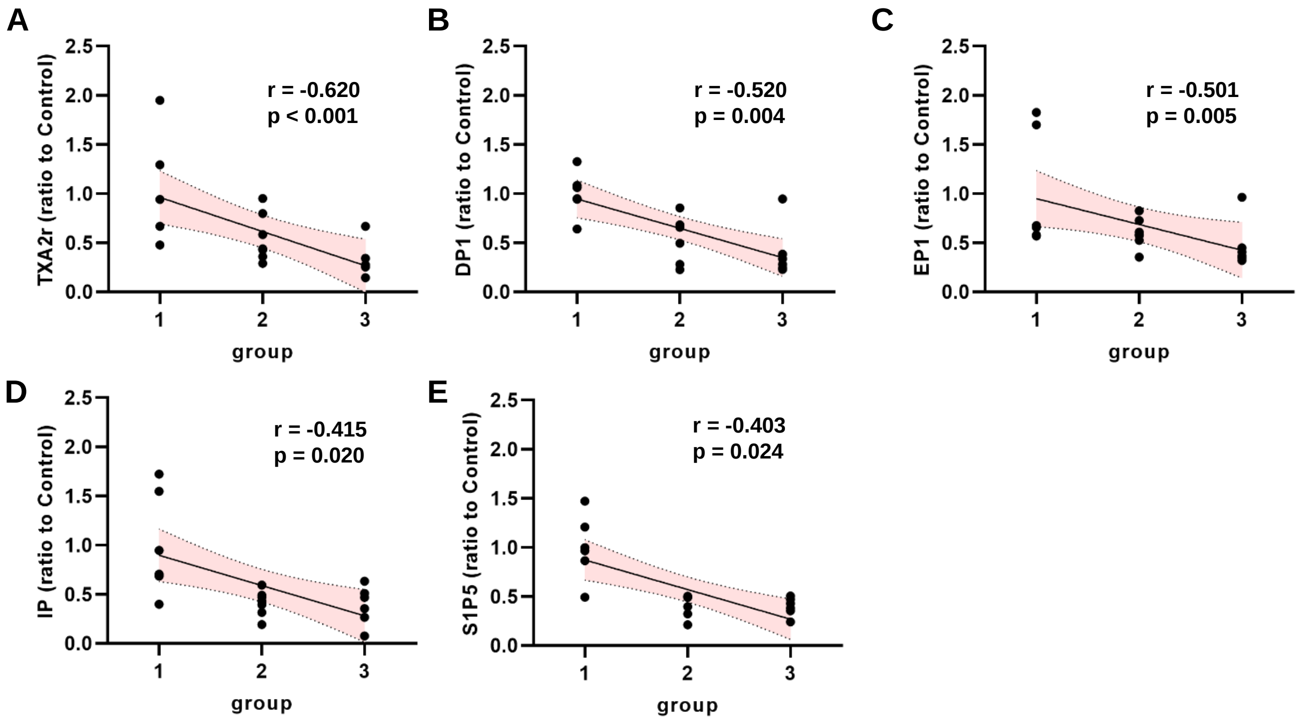

Supplemental Figure S3. Correlations between bioactive lipid receptors and the diagnostic group. The significant correlations of bioactive receptor expressions and the diagnostic group are shown.

Supplemental Figure S4

Loading 1

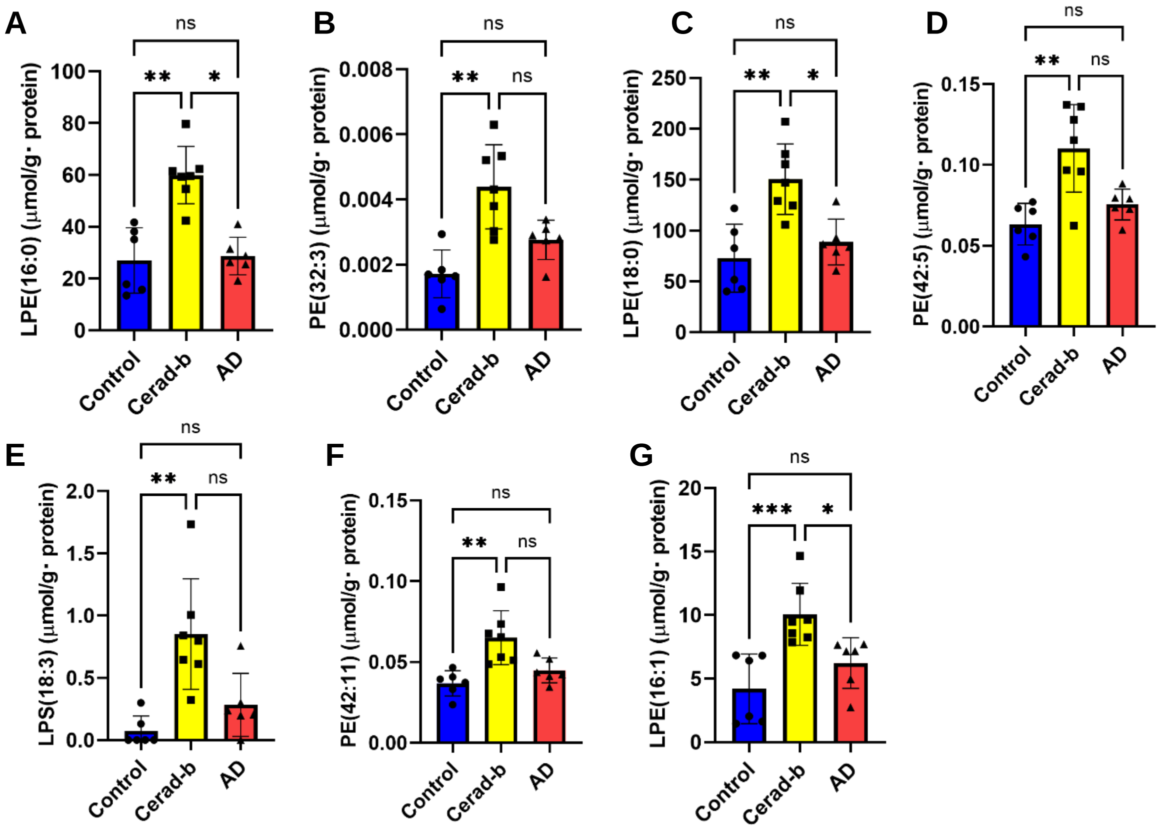

Loading 2

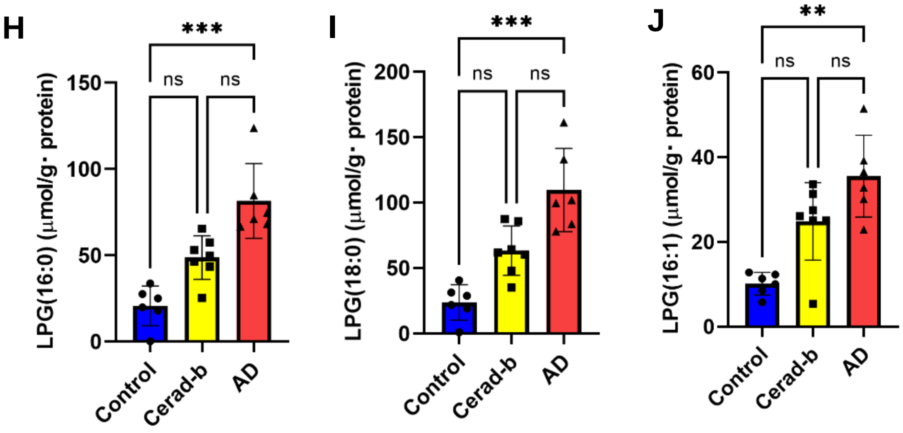

Supplemental Figure S4 Modulations of bioactive lipid levels in the brain in AD and Cerad-b. The levels of bioactive lipids that contributed substantially to component 1 (A–G) and component 2 (H–J) in Figure 6 are shown. *P<0.05, **P<0.01.

Supplemental Figure S5

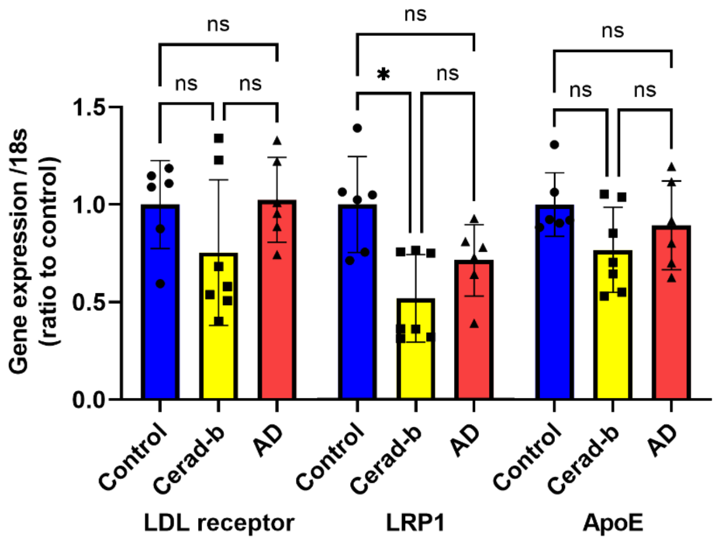

Supplemental Figure S5. Modulations of LDL receptor, LRP1, and ApoE mRNA levels in the brain of AD and Cerad-b.

The mRNA levels of LDL receptor, LRP1, and ApoE mRNA levels in the brain samples were determined with real-time PCR. The mRNA levels of genes were adjusted to the expression level of 18s as the internal standard. *P<0.05.
